# Core set approach to reduce uncertainty of gene trees

**DOI:** 10.1186/1471-2148-6-41

**Published:** 2006-05-20

**Authors:** Takahisa Okabayashi, Yasuhiro Kitazoe, Hirohisa Kishino, Teruaki Watabe, Noriaki Nakajima, Yoshiyasu Okuhara, Samantha O'Loughlin, Catherine Walton

**Affiliations:** 1 Center of Medical Information Science, Kochi Medical School, Kochi University, Okoho-cho, Nankoku, Kochi 783-8505, Japan; 2 Graduate School of Agriculture and Life Sciences, University of Tokyo, Yayoi, Bunkyo, Tokyo, 113-8657, Japan; 3 Faculty of Life Sciences, University of Manchester, Manchester, M13 9PT, UK

## Abstract

**Background:**

A genealogy based on gene sequences within a species plays an essential role in the estimation of the character, structure, and evolutionary history of that species. Because intraspecific sequences are more closely related than interspecific ones, detailed information on the evolutionary process may be available by determining all the node sequences of trees and provide insight into functional constraints and adaptations. However, strong evolutionary correlations on a few lineages make this determination difficult as a whole, and the maximum parsimony (MP) method frequently allows a number of topologies with a same total branching length.

**Results:**

Kitazoe et al. developed multidimensional vector-space representation of phylogeny. It converts additivity of evolutionary distances to orthogonality among the vectors expressing branches, and provides a unified index to measure deviations from the orthogoality. In this paper, this index is used to detect and exclude sequences with large deviations from orthogonality, and then selects a maximum subset ("core set") of sequences for which MP generates a single solution. Once the core set tree is formed whose all the node sequences are given, the excluded sequences are found to have basically two phylogenetic positions on this tree, respectively. Fortunately, since multiple substitutions are rare in intra-species sequences, the variance of nucleotide transitions is confined to a small range. By applying the core set approach to 38 partial *env *sequences of HIV-1 in a single patient and also 198 mitochondrial COI and COII DNA sequences of *Anopheles dirus*, we demonstrate how consistently this approach constructs the tree.

**Conclusion:**

In the HIV dataset, we confirmed that the obtained core set tree is the unique maximum set for which MP proposes a single tree. In the mosquito data set, the fluctuation of nucleotide transitions caused by the sequences excluded from the core set was very small. We reproduced this core-set tree by simulation based on random process, and applied our approach to many sets of the obtained endpoint sequences. Consequently, the ninety percent of the endpoint sequences was identified as the core sets and the obtained node sequences were perfectly identical to the true ones.

## Background

Molecular phylogeny at the intraspecific level has proven to be very useful for a variety of studies, such as estimating the genealogical relationships among genes, determining the common ancestor of a group of organisms, evaluating the development of drug resistance in infectious diseases, detecting positive selection for immune escape, and predicting future trends of diseases [1-7]. The sequences within a species are similar each other compared with those from different species. Therefore, it is generally expected that the pairwise difference is a good approximation to the evolutionary distance, and the estimated phylogeny by existing methods (maximum parsimony (MP) [8,9], statistical parsimony (SP) [13-17] and maximum likelihood (ML) [11,12]) is reliable and has little uncertainty. However, there appear sometimes abnormal phenomena in which MP picks up a prohibitive number of equally parsimonious trees [13]. Such phenomena suggest the presence of strong evolutionary correlations among different lineages. Then, a nested analysis approach [18-24] makes a complicated network mapping in a nested analysis and makes it impossible to trace the evolutionary history. In this situation, when the approximation of the pairwise difference is well satisfied as a whole, we can expect that the above abnormal phenomena are caused by a small number of sequences in a given dataset.

Recently, Kitazoe et al. [25,26] proposed multidimensional vector-space (MVS) representation in which the estimated pairwise distances can be reproduced by using Pythagorean theorem. When a distance matrix is compatible with a tree structure, sequences are expressed by the composites of branch vectors that are orthogonal among others. Therefore, MVS representation enables to measure the deviation from the tree structure which subsists in the initial pairwise differences. In this paper, this measured deviation serves as a unified index in the core set approach developed below.

We principally select a maximum subset ("core set") of sequences for which MP generates a single solution, by excluding sequences with large values of the deviation index, and we can uniquely determine all the node sequences of the core set tree. Here, we assume that this core set tree is robust and preserved by insertion of the excluded sequences. This assumption seems to be reasonable when the number of the excluded sequences is small. The core set approach makes the reason of the exclusion clear, and therefore makes it possible to estimate the nucleotide transitions within a small range of uncertainty. Indeed, we found through applications that the excluded sequences without recombination have two phylogenetic positions, respectively. A more detailed procedure of the core set approach is documented in the followings:

We first select a maximum number ("primary core (P-core) set") of sequences (the procedure (a) in Methods) whose distance sub-matrix gives no deviation from additivity of evolutionary distances. Existing methods (MP, SP, ML and NJ) produce the same single tree as the P-core set tree. Second, the excluded sequences are added on the P-core set (the procedure (b) in Methods). This insertion provides a maximum set ("secondary core (S-core) set") of sequences for which MP produces a single tree, and the S-core set comprises a global mainframe of the final tree structure. However, long branching attractions may sometimes prevent a further restore of the excluded sequences into the S-core set. In such a case, we divide the S-core set into several subgroups and the two procedures of exclusion and insertion are repeated in the subgroups on the additional condition of preserving the S-core set tree structure.

We apply the core set method to partial *env *sequences of HIV-1 (C2-V5), to demonstrate how our approach realizes reasonable tree building. We next employ the method for a phylogenetic analysis of mitochondrial sequences of *Anopheles dirus*, which characterize a simultaneous radiation pattern. Then, we perform simulation studies and confirm that our core-set is mostly a large proportion of the original dataset and that the inferred tree for the S-core set is consistent with the true tree of the whole sequences.

## Results

### 1) HIV phylogeny inference by constructing only a S-core set tree

**Figure 1:**
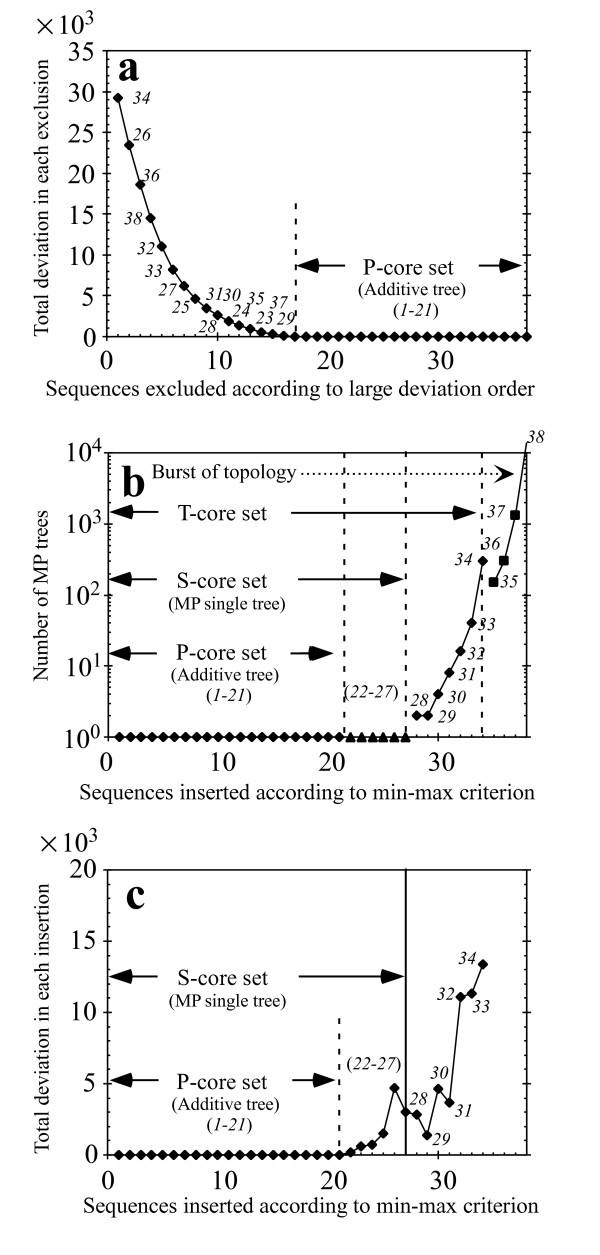
**Tree-building process by the core set approach**. Figure **a **shows the excluding process of sequences in the order of the largest deviation from additivity. Finally, the 21 sequences remained for the P-core set formed by the single substitution (solid circle). The first 27 sequences give the S-core set which MP makes a unique tree (solid triangle in Fig. **b**). The first 34 sequences give the T-core set tree formed by using both the min-max criterion and the condition of preserving the S-core set tree, though MP shows 300 topologies (solid diamond in Fig. **b**). The inclusion of the remaining 4 sequences gives rise to an explosion of topology (the solid squares in Fig. **b**). Figure **c **follows the W_i _values of sequences to be inserted into the core set by using both the min-max criterion.

We applied our method to partial *env *sequences of HIV-1 (C2-V5) that were obtained in a longitudinal study [27] of many patients. We analyzed a subgroup of 38 nucleotide sequences from the patient-1, which comprise an obvious monophyletic tree and are separated from the other subgroups in MP consensus tree. Hence, there unlikely exist substitutions which make phylogenetic inference much difficult and this data set provided a good example to demonstrate the validity of our method (these sequences and their accession numbers are listed in Additional file 1 1). We first selected a P-core set of sequences, which made the total deviation index W (Eq. (3) in Methods) equal to zero by performing the procedure (a) in Methods. The sequence number *1*–*21 *in Fig. 1b stands for this P-core set member. Here, standard methods such as NJ, MP and SP provided the same tree, because there were no multiple substitutions in the P-core set. We next tried to incorporate the excluded 17 sequences sequentially into the P-core set by performing the procedure (b) in Methods. As a result, the sequence number *22*–*27 *were further deposited on the P-core set tree, and a S-core set tree of the total 27 sequences was obtained (Fig. 1b). Here, the S-core set produced the set of maximum number of sequences for which MP provides a single tree. Indeed, we confirmed that there is no other subset with 27 sequences except for our S-core set which has a single MP tree by examining all possible combinations between 27 (and more) sequences and the remaining sequences. The number of sequences in the other subsets was always less than 27. We further performed the procedure (c) that decomposes the S-core set into subgroups. However, we found that there was no more sequence which could be added by the criterion of procedure (c). In this way, our final tree was constructed by only once performing the two procedures (a) and (b) in Methods. We here note by investigating all possible combinations of two groups that the P-core set of 21 sequences is a maximum number set that satisfies the additivity but the second P-core set is given by replacing the 16^th ^sequence by the 22^th ^sequence. With starting this second set, however, the original S-core set of 27 sequences did not change.

**Figure 2:**
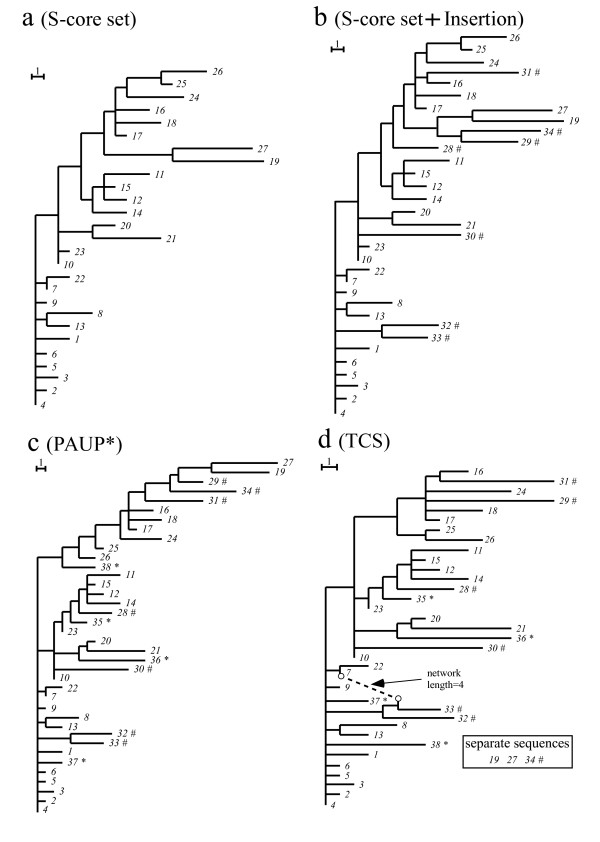
**Comparison between the core set approach and standard methods**. (a) The core set tree in which MP gives a single solution. (b) The final tree formed by preserving the core set tree and without any alternative phylogenetic positions in the inclusion of the excluded sequences. (c) One of MP tree (PAUP*) formed without preserving the core set tree. (c) SP tree (TCS) formed without preserving the core set tree.

As an extension of our core-set approach, we could add further seven sequences on the S-core set (sequences *28*–*34*, designated # marks (Fig. 2b)), which could be given unique phylogenetic positions on the condition of preserving this tree by the criterion of procedure (b). Consequently, the T-core set was constructed (procedure (b) in Methods). Here, MP method provided multiple solutions of phylogeny (300 tree patterns with MP software package PAUP* [9]) (Fig. 1). A further inclusion of sequences *35*–*38 *with larger deviations (denoted *) generated an explosion of topologies (14529 tree patterns) with PAUP*. Some of these patterns disrupted even the core set tree (Fig. 2c). SP tree (TCS version 1.13 software package) [28] inferred from the S-core set (*1*–*27*) separated two sequences (*19 *and *27*) from the core set, and the inclusion of all sequences disrupted the S-core set tree (Fig. 2d). One way to address these topological uncertainties of MP and SP is to first fix the topology using ML, and then to determine the node sequences using MP [29]. However, ML + PAUP* also disrupted the core set (Additional file 2). The topology and node sequences were calculated with ML, software package PHYLIP (ML) [12] and PAUP*, respectively.

### 2) Mosquito phylogeny inference by constructing local core-set trees

**Figure 3:**
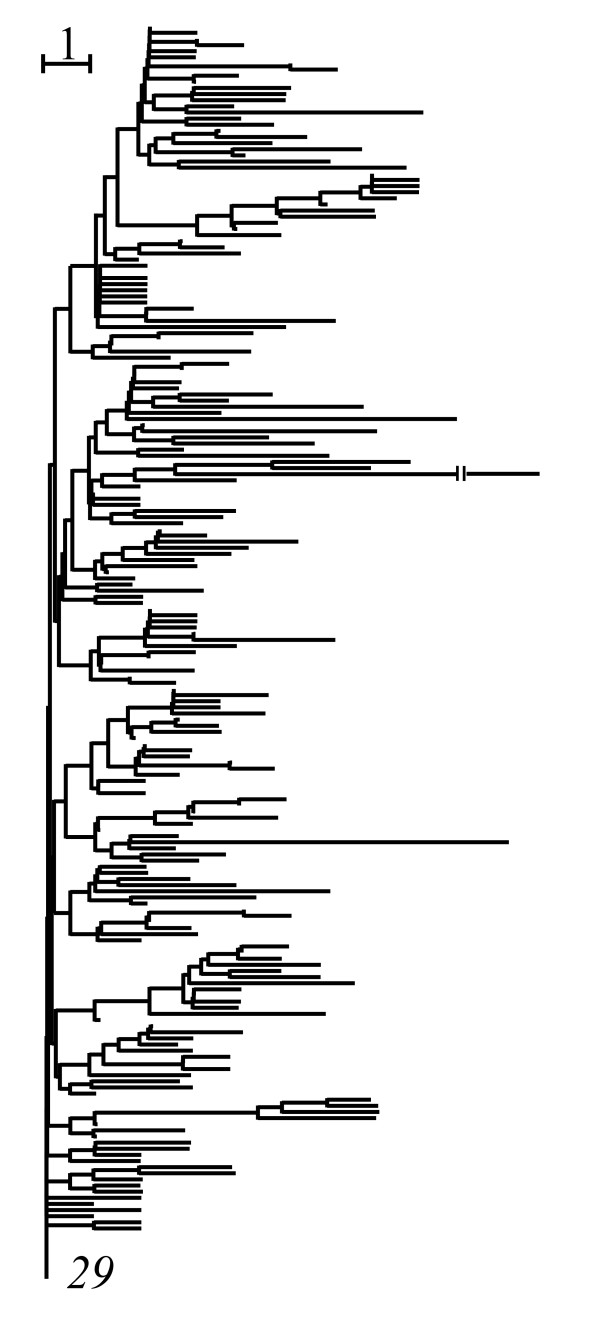
**NJ tree of *A. dirus *mosquitoes with 198 haplotypes and 1537 sites. **The tree was obtained by using the pairwise differences (the *29th *haplotype was taken as the outgroup). It was considerably distorted by evolutionary correlations, since the branch lengths should be integers.

The core set approach was applied to a data set of mitochondrial COI and COII DNA sequences of *Anopheles dirus *species A and D of Southeast Asia [30,31]. NJ tree of which the outgroup was the *29th *haplotype was highly distorted and many of branching lengths on the tree represented smaller numbers of single substitutions (Fig. 3). Such abnormal phenomena were due to strongly evolutionary correlations among many different branches.

**Figure 4:**
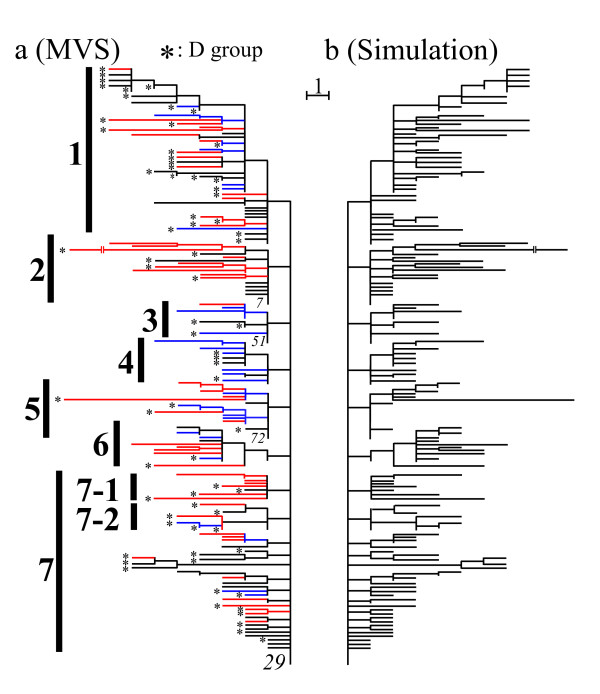
**Core set tree of *A. dirus *mosquitoes and evolutionary simulation of this tree**. (a) The black lines represent branches for the global core set (71 haplotypes), which satisfies the orthogonality perfectly. The global core set was decomposed into nine groups (the vertical bold lines). Here, each group had the *29th *haplotype in common. The blue lines denote the 35 haplotypes of the local P-core set, while the red lines show the 58 haplotypes of the local S-core set. The remaining 34 haplotypes were excluded because of the shared-site uncertainty explained in Fig. 7. (b) We simulated a Markov process of sequence evolution, which resulted in the same phylogenetic tree as the core-set tree (a). Application of the core set approach to a set of the terminal sequences retained 154 sequences of the whole 164 sequences as the global core-set member without requiring any local core-set trees. The obtained node sequences were perfectly identical to the true ones. The global core set produced a unique MP tree. The inclusion of the remaining 10 sequences allowed MP to propose a number of topologies (804).

We first constructed a global core set (S-core set) consisting of 71 haplotypes by using the procedures (a) and (b) in Methods (the black lines of Fig. 4a). The tree structure of this core set represents a simultaneous radiation (star-like) pattern centered on the *29th *haplotype. We next decomposed the global core set into subgroups by using the procedure (c) in Methods. The subgroups obtained by this procedure are shown by the vertical bold lines of Fig. 4a. Here, the *29th *haplotype was included in all groups as their common ancestor. The procedure (a) was then applied to the individual subgroups, and the total 35 haplotypes were added in the seven subgroups (the blue lines of Fig. 4a). Fifty-eight of the remaining 92 haplotypes were inserted into each of the obtained local P-core sets by using the procedure (b) (the red lines of Fig. 4a). The second, third, and fourth columns of Table 1 give the haplotype numbers corresponding to the global core set, the local P-core sets, and the local S-core sets in each group, respectively. As a result, our final tree consists of 164 haplotypes (Fig. 4a). This tree has a finely resolved structure with definite number of substitutions on each branch, in contrast to NJ tree (Fig. 3). The asterisks denote the haplotypes of *A. dirus *D, whereas those of *A. dirus *A are unmarked. Haplotypes of *A. dirus *species A and D are intermingled across the tree. This can be interpreted as resulting from the introgression of mtDNA between the two *A. dirus *species. Details of the biological interpretations will be reported by O'Loughlin et al. [30].

**Table 1:** Numbers of haplotypes incorporated by the three procedures (a)-(c) of the core set approach.

Group No.	Global core	Local P-core	Local S-core	Total
1	26	8	15	49
2	8	0	10	18
3	4	4	1	9
4	6	7	0	13
5	4	6	7	17
6	6	3	5	14
7	23	7	20	51

Total	71	35	58	164

Insertion rate(%)	35.9	18.7	28.2	82.8

The second column includes the *29th *haplotype. The third and forth columns give only the haplotype number additionally inserted.

Since we could definitely determine the node sequences in the final tree (Fig. 4a), we estimated the frequencies of nucleotide substitutions. We counted all the nucleotide substitutions from the *29th *haplotype (radiation centre) toward the endpoints. Of the 1537 sites, 176 sites showed nucleotide substitutions (Table 2). Of these, 55 sites had undergone multiple substitutions within the genealogy of the final tree. On the other hand, thirty-one sites had undergone non-synonymous changes, among which only five sites had experienced multiple substitutions. The multiplicity of the 176 variable sites averaged out to 1.8 because there were 312 substitutions in total. However the multiplicity was not distributed homogeneously among the variable sites. The largest number of multiple substitutions at one site which we observed was 15 (Table 2). Of these 312 substitutions, 269 were transitions and 43 were transversions (Table 3a). Non-synonymous changes comprised 10% of the total, and T→C and A→G transitions predominated. This feature became more obvious at small multiplicities with n < 4 (Table 3b). We finally estimated the number of nucleotide transitions by incorporating the 19 sequences excluded from the core set (Table 4a). Although these sequences have two phylogenetic positions, respectively, the collection of all possible patterns provided a small fluctuation in each transition matrix, as seen in this Table.

**Table 2:** Numbers of sites with single and multiple substitutions.

Number of substitutions at a site	1	2	3	4	5	6	7	8	9	15	Total
Number of sites (nucleic acids)	121	26	14	3	5	2	1	1	2	1	176
Number of sites (amino acids)	26	4	1	0	0	0	0	0	0	0	31

**Table 3:** Frequency of nucleotide substitutions.

a (Inclusive)					
	A	C	G	T	Total

A	0	7	81	14	102
C	2	0	0	45	47
G	52	0	0	0	52
T	11	91	9	0	111

Total	65	98	90	59	312

b (n < 4)					

	A	C	G	T	Total

A	0	4	57	12	73
C	1	0	0	32	33
G	18	0	0	0	18
T	8	74	9	0	91

Total	27	78	66	44	215

This shows that T→ C and A→ G transitions predominated (Table 3a), especially in the case of small numbers (n < 4) of multiple substitutions (Table 3b).

**Table 4:** Frequency of nucleotide transitions.

a					
	A	C	G	T	Total

A	-	7.5 ± 0.5(7)	94.0 ± 1.0(81)	14.5 ± 0.5(14)	116.0 ± 1.2(102)
C	3.0 ± 0.0(2)	-	0.0 ± 0.0(0)	56.0 ± 1.0(45)	59.0 ± 1.0(47)
G	69.0 ± 1.6(52)	0.0 ± 0.0(0)	-	0.0 ± 0.0(0)	69.0 ± 1.6(52)
T	13.0 ± 1.0(11)	108.0 ± 1.2(91)	10.0 ± 0.0(9)	-	131.0 ± 1.4(111)

Total	85.0 ± 1.4(65)	115.5 ± 1.3(98)	104.0 ± 1.0(90)	70.5 ± 1.1(59)	375.0 ± 0.0(312)

b					

	A	C	G	T	

A	-	6.5 ± 0.4(6.9)	81.0 ± 0.5(79.4)	12.5 ± 0.4(13.7)	
C	5.1 ± 0.1(4.3)	-	0.0 ± 0.0(0)	94.9 ± 0.1(95.7)	
G	100.0 ± 0.0(100)	0.0 ± 0.0(0)	-	0.0 ± 0.0(0)	
T	9.9 ± 0.7(9)	82.4 ± 0.7(82)	7.6 ± 0.1(9)	-	

Table 4. The frequency of nucleotide transitions (a) and the relative frequency (%) (b). The number of brackets denotes the frequency of nucleotide transitions in only the core set tree.

### 3) Efficacy of the core set approach by evolutionary simulation of random process

To examine the efficacy of the core set approach, we simulated a Markov process of sequence evolution, which resulted in the same phylogenetic tree as the core-set tree derived from the 164 mitochondrial COI and COII DNA sequences of *Anopheles dirus *(Fig. 4a). Here, the root sequences were randomly generated according to the nucleotide frequency distribution of the *29th *haplotype in Fig. 4a. Each nucleotide substitution was randomly generated according to the weight given in Table 3a. Since non-synonymous substitutions were rare in the above analysis, we only changed the third codon positions. Although the site heterogeneity of the evolutionary rate was neglected for simplicity, there is more chance of multiple substitutions because we kept the branch lengths. Application of the core set approach to 10 sets of the terminal sequences retained 90 % of the whole sequences as the global S-core set member and did not require any local core-set trees because of random process without strong evolutionary correlations (long-branch attractions). The global S-core set produced a unique MP tree which is consistent with the simulated tree. Furthermore, the estimated sequences at the internal nodes of the S-core set were perfectly identical to the true ones. Figure 4b illustrates the global S-core set tree in one event (the endpoint sequences of this event are listed in Additional file 3). The inclusion of the remaining 10 % sequences allowed MP to provide a number of topologies around 800. This implies that the only sequences with phylogenetic uncertainty were excluded by the core set approach.

## Discussion

The core set approach first determines a set of the sequences (P-core) that form the tree structure without multiple substitutions by using only the total deviation index. The approach next tries to sequentially insert the excluded sequences into the P-core set tree, according to the min-max criterion. If these sequences have the unique phylogenetic positions on the P-core set, they are incorporated into the P-core set as the S-core set. The min-max criterion is useful for determining order of the insertion. In fact, in the present demonstration of the HIV sequences (Fig. 1), the S-core set was formed without any missing by just stopping the insertion when MP gives multiple solutions. In this way, the core-set approach takes into explicit account of the tree-building process to construct a consistent and robust tree.

**Figure 5:**
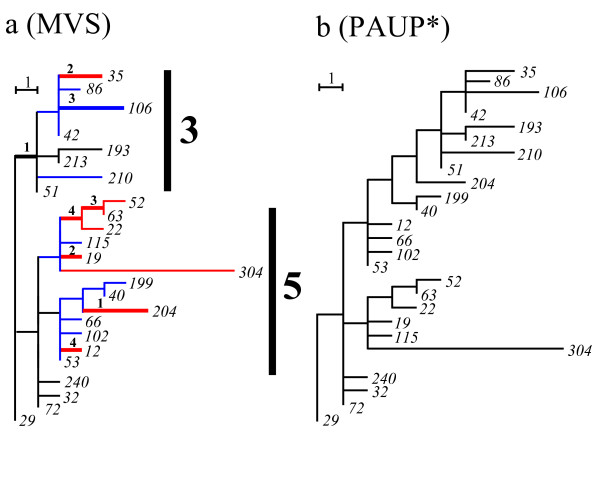
**Core set tree compared to MP tree**. We considered the 25 haplotypes of groups 3 and 5 in Fig. 4a. PAUP* yielded 15 different branch patterns on the maximum parsimony criterion. One of them gave the same result as the core set approach (Fig. 5a), while another of them gave a very different pattern from our result (Fig. 5b). The bold lines indicated by block letters in Fig. 5a denote parallel changes of four pairs.

A systematic analysis of the mosquito mitochondrial DNA data set also showed that the maximum parsimony criterion was insufficient to produce a single tree structure. To demonstrate this lucidly, we consider only two groups, 3 and 5 in Fig. 4a, which consist of nine and 17 haplotypes including the *29th *haplotype, respectively. The 25 haplotypes of the two groups were analysed by using PAUP*. The analysis generated 15 different branch patterns, five of which were consistent with the branch pattern of the global core set. Only one of these five trees corresponded to our result (Fig. 5a). The other trees showed very different branch patterns from ours, as illustrated in Fig. 5b. Using our approach, we first selected the global core set of seven sequences (black lines), then the local P-core set of 10 sequences (blue lines), and then eight sequences (red lines) with the local S-core set. The fifteen topologies generated by PAUP* were the result of parallel changes between four sites and four branching pairs in the two groups (see the bold lines of Fig. 5a). The application of PAUP* to all 164 haplotypes of Fig. 4a generated more than 100,000 topologies. In this way, the core set approach makes neighbor-joining trees robust by avoiding evolutionary correlations with other groups.

**Figure 6:**
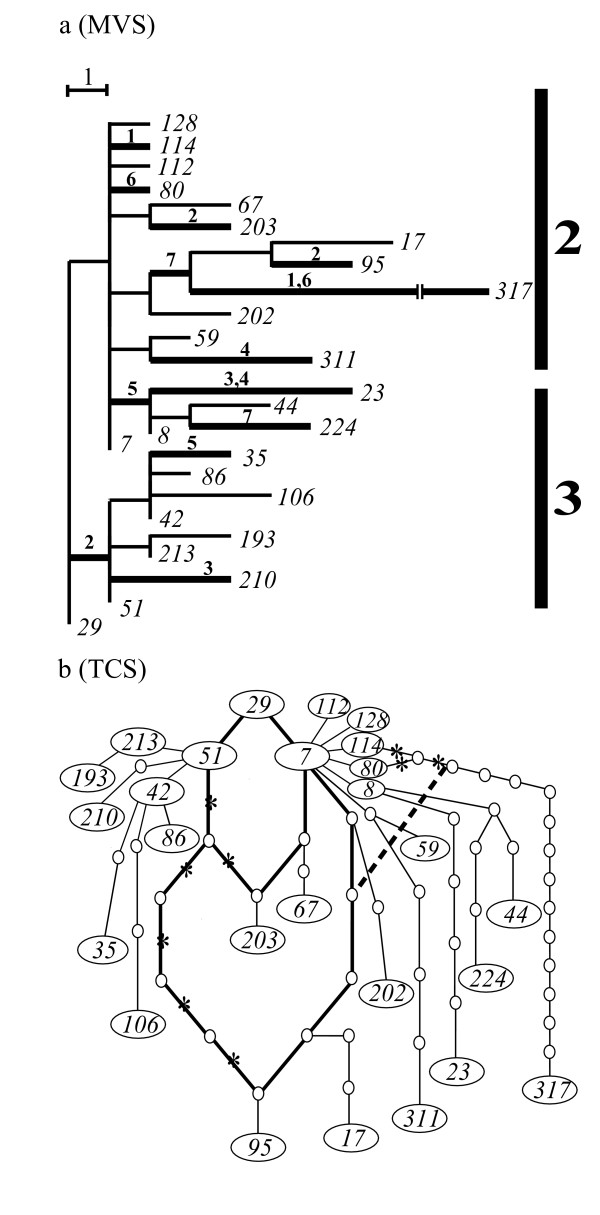
**Core set tree compared to SP tree. **We considered the 26 haplotypes of groups 2 and 3 in Fig. 4a. TCS had complicated loops in the tree (Fig. 6b). Removal of the branches marked with asterisks and changing the dotted line into a solid line gave our result (Fig. 6a), which was also uniquely reproduced by PAUP*. The occurrence of loops could be explained by parallel changes. The bold lines in Fig. 6a denote parallel changes of six pairs and one triplet.

By contrast, SP reduced the topological uncertainty of PAUP* to a large extent. On the other hand, the network representation made it difficult to follow the evolutionary process strictly. Complicated network mapping was demonstrated by applying TCS to the 26 haplotypes of groups 2 and 3 in Fig. 4a (Fig. 6b). The reticulated pattern can be explained by parallel changes (six pairs and one triplet of branches, shown with bold lines in Fig. 6a) mainly between the two groups. In Fig. 6b, removal of the branches marked with asterisks and changing the dotted line to a solid one produces our result (Fig. 6a). Note that PAUP* reproduced the same unique solution as ours. Applying the TCS to all the haplotypes of Fig. 4a generated a much more complicated reticulated genealogy. We applied ML + PAUP* to the 164 haplotypes in Fig. 4a. The result obtained was rather different from that of the core set approach. We demonstrate a typical example of these differences using the two sub-groups 7–1 and 7–2 in Fig. 4a (Additional file 4).

**Figure 7:**
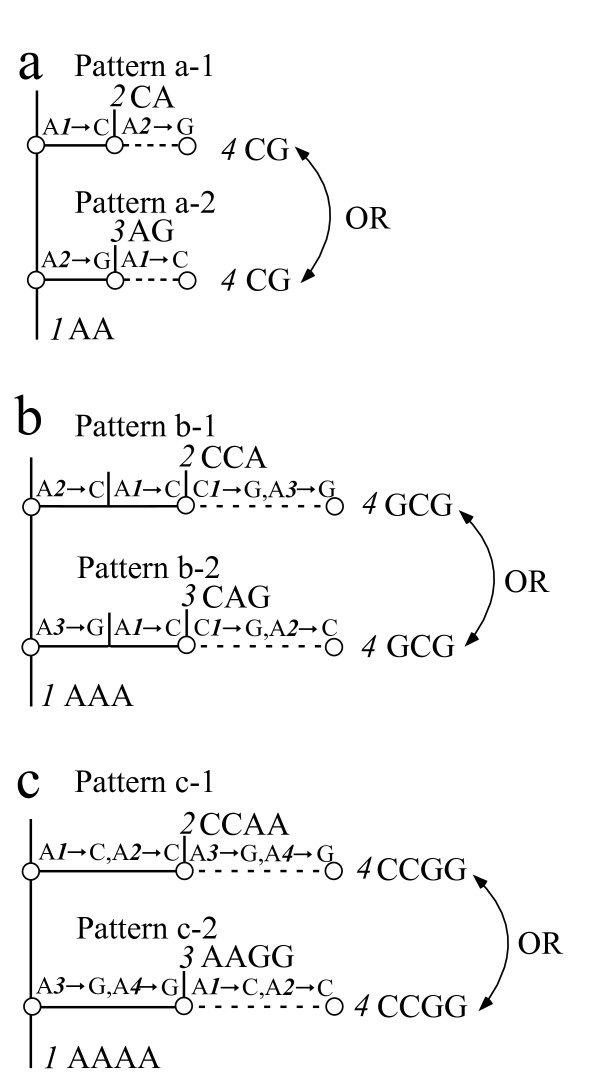
**Three alternative branch patterns caused by evolutionary correlations**. The branching position of the *4th *sequence depends on nucleotide substitutions between the *1st *sequence and the *2nd *and *3rd *sequences, and cannot be uniquely determined by the maximum parsimony criterion. Figure 7a shares one of two sites. Figure 7b shares two of three sites. Figure 7c shares two of four sites. Here, A *n*→ C denotes a substitution from A to C at the *n th *site.

**Figure 8:**
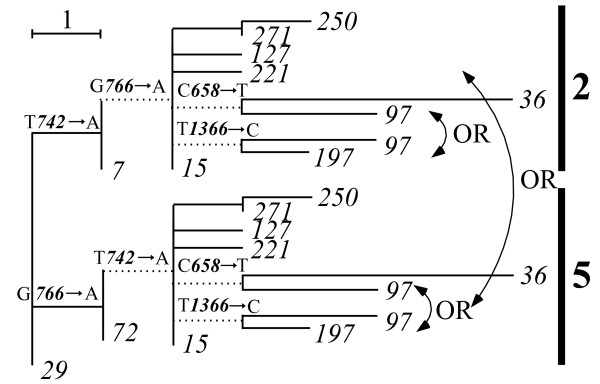
**Typical example of two phylogenetic positions in mosquitoes**. Example of alternative phylogenetic positions caused by the shared-site uncertainty illustrated in Fig. 7a. It remains ambiguous whether the *15th *haplotype should be branched from the *7th *haplotype (node) in group 2 or from the *72nd *haplotype (node) in group 5. This uncertainty was a major influence on the phylogeny, because the *72nd *haplotype became the ancestor of the *250th*, *271st*, *127th*, *221st*, *36th*, *97th*, and *197th *haplotypes. Here, the last three haplotypes yielded an additional uncertainty of their locations. Here, A*n*→C denotes a substitution from A to C at the *n *th site.

Of the 198 haplotypes, 34 had alternative phylogenetic positions, as discussed in Methods. Thirty-three haplotypes gave the one-site sharing pattern of Fig. 7a, and one haplotype gave the two-site sharing pattern of Fig. 7c. Figure 8 illustrates a typical example of uncertainty whether the *15th *haplotype should be inserted into the *7th *haplotype (node) in group 2 or into the *72nd *haplotype (node) in group 5. This uncertainty exerts a strong influence on the phylogeny, because the *15th *haplotype becomes the ancestor of the *250th*, *271st*, *127th, 221st*, *36th*, *97th*, and *197th *haplotypes, and the last three of these haplotypes confer a further ambiguity because the *97th *haplotype produces the one-site sharing pattern.

The present core set approach implicitly assumes the condition that the global core set includes a majority of sequences and constructs a main frame of tree structure. When we cannot assume this condition, we decompose the total sequences into some groups, by using the criterion of the 100 % branching resolution in MP consensus tree. Then, the present core set approach is applied to each group. The phylogeny among the groups is determined so that the branching lengths to connect them may be minimized. Details of this subject will be discussed elsewhere.

## Conclusion

The global core-set member was first selected from the complete set of sequences by the procedures (a) and (b) in Methods, and comprised the mainframe for the further tree-building construction. The local core-set trees were second constructed by preserving this mainframe. Only the sequences with alternative phylogenetic positions finally remained out of the tree. The core-set approach is assigned so that MP provides single trees for the global core-set and for each of local core-sets. Hence, MP allows multiple solutions for our final set of sequences given by combining the local core-sets. This approach provides a definite and unique tree structure without relying upon probabilistic models of sequence evolution, by excluding the sequences casusing phylogenetic uncertainty. Such a set of well-defined evolutionary pathways will provide increased power in making inferences of demographic history, and be useful in clarifying the precise mechanisms of molecular evolution. One of our next tasks is to detect definitely the sequences associated with recombination [32,33], by systematically analysing data sets of many HIV-1 patients [27].

## Methods

### General idea of the core set approach

There is generally considerable uncertainty in the inference of a phylogenetic tree. However, in the study of molecular evolution within a population, it may be possible to make more precise inferences because the intraspecific variability of sequences is much less than the interspecific ones. Unfortunately, the existing procedures of phylogenetic inference do not fully incorporate this feature.

In our core set approach, we collect a large subset of sequences from a population, for which a reliable phylogenetic tree can be uniquely estimated. We take the infinite allele model, which neglects multiple substitutions at a nucleotide site, as a first approximation to the molecular evolution within a population. Therefore, we start by finding a subset for which the matrix of pairwise differences (numbers of different sites) is compatible with some unknown tree. This compatibility is measured by the index of deviation from the orthogonality, which we developed via MVS representation of sequences [26]. It is possible to calculate the deviation without constructing a tree. We sequentially exclude sequences that contribute greatly to the deviation until the deviation reaches the value of zero. This procedure provides a maximum set of sequences for a unique tree without multiple substitutions, which is easily estimated by any methods of phylogenetic inference. The second procedure sequentially inserts the excluded sequences into the obtained core-set tree until MP gives multiple trees, to obtain a global core set. The above-mentioned two procedures are repeated in subsets of the global set to obtain a further inclusion of the excluded sequences by removing long branching correlations.

### MVS representation of phylogeny and index of deviation from additivity

**Figure 9:**
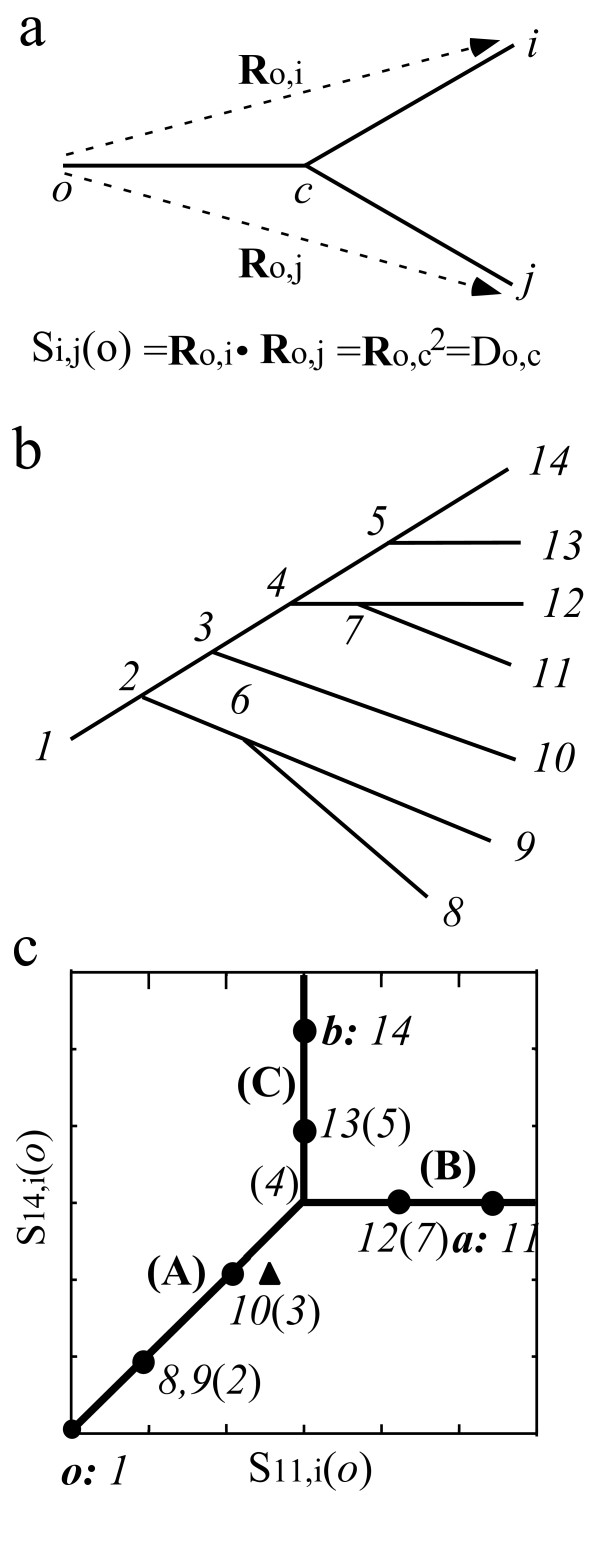
**The MVS representation of a branching pattern**. The distance D_o, c_between *o *and *c *is given by the inner product, S_i, j_(*o*), of two spatial vectors, R_o, i _and R_o, j_in MVS (Fig. 9a). The branching pattern of Fig. 9b and corresponding MVS representation of Fig. 9c in terms of S_11, i_(*o*) and S_14, i_(*o*). Here, the triplet of probes *o *= *1*, *a *= *11*, and *b *= *14 *were used. The endpoints (*8*, *9*), *10*, *12*, and *13 *are degenerated into the positions of the nodes *2*,*3*,*7*, and *5*, respectively. When the pairwise differences satisfy orthogonality, the scatter plots are placed on the orthogonal lines A, B and C. Convergent evolution between the two branches (*3*→ *10*) and (*7*→ *11*) underestimates the pairwise difference between *10 *and *11*, causing a deviation of point *10 *from the orthogonal line A, which moves it to the position of the triangle.

The orthogonal feature of the spatial branch vectors in MVS is essential for resolving the tree structure. Because MVS cannot be directly visualized, we use the spatial vectors of some sequences as probes ("search vectors") and investigate how other sequences are branched around the probes. A sequence *o *is selected as one of the probes and placed at the coordinate origin of MVS, from which the spatial positions, R_o, i_, of other sequences *i *are measured. The inner product, S_i, j_(*o*), of two vectors, R_o, i _and R_o, j_, gives the branch length from the origin *o *to the most recent common ancestor *c *of sequences *i *and *j*, if the branch vectors are orthogonal (Fig. 9a). This is proved by the relation S_i, j_(*o*) = R_o, i_·R_o, j _= (R_o, c _+ R_c, i_)·(R_o, c _+ R_c, j_) = R_o, c_^2 ^= D_o, c_, sinceR_o, c_⊥R_c, j, _R_o, c_⊥R_c, i,_and R_c, i_⊥R_c, j_. Here, D_i, j_is the pairwise difference between the two sequences *i *and *j *and is define das equal to R_i, j_^2^. The branch length can be rewritten as S_i, j_(*o*) = R_o, i_R_o, j _cos(θ_i, j_), with the angle θ_i, j _between the vectors R_o, i _and R_o, j_. Using the cosine theorem (R_i, j_^2 ^= R_o, i_^2 ^+ R_o, j_^2^-2R_o, i _R_o, j_cos(θ_i, j_)) about the triangle (*o*, *i*, *j*) gives the equation,

S_i, j_(*o*) = (D_o, i _+ D_o, j _- D_i, j_)/2.     (1)

MVS representation of a tree is expressed using two other probes, *a *and *b*. We make a scatter diagram in terms of S_i, a_(*o*) and S_i, b_(*o*), which are taken to be the x and y values, respectively. Figure 9 illustrates the relationship between the standard tree expression (Fig. 9b) and the corresponding MVS representation (Fig. 9c). In Fig. 9c, *o = 1*, *a = 11*, and *b = 14 *were taken as a triplet of probes. Here, all sequences (solid circles) lie on the three lines A, B, and C (hereafter called the "orthogonal lines"), and fulfil the following equations for orthogonality: S_11,8_(*1*) *= *S_11,9_(*1*) = S_14,8_(*1*) *= *S_14,9_(*1*) = D_1,2_; S_11,10_(*1*) *= *S_14,10_(*1*) = D_1,3_; and S_11,13_(*1*) = S_11,14_(*1*) = S_14,12_(*1*) = D_1,4_. Endpoints (8–10), 12 and 13 are arrayed along the orthogonal lines in the order of branching from the probes, because their branch vectors are orthogonal to the probe vectors R_1,11 _and R_1,14_. Points (*8*–*10*) outside the common ancestor *4 *of the probes *11 *and *14 *lie on the diagonal line A. Point *12*, closest to probe *11*, is located on line B, and point *13*, closest to probe *14*, is located on line C. Points *8 *and *9 *have been degenerated into the position of node *2*. The degeneracy can be resolved using other triplets of probes. In this way, the tree structure is analysed using a variety of triplets.

For a practical inference of the phylogeny, the pairwise distances must be estimated initially. Because the core set approach focuses on describing the evolutionary process within a species, the initial distances can be approximated well by the pairwise differences. Any multiple substitutions that have occurred on the evolutionary pathway between the two sequences cause an underestimate of the corresponding pairwise difference, which violates orthogonality. For example, when we consider parallel changes between the two branches (*3*)–(*10*) and (*7*)–(*11*), MVS representation expresses these changes as a deviation of endpoint *10 *from the diagonal line A, as shown by the triangle in Fig. 9c.

The deviation of a sequence *i *is defined as the distance from the position of this sequence to the nearest orthogonal line in each triplet (*o*, *a*, *b*). Because S_a, b_(*o*) gives the y value of the line B and the x value of line C in Fig. 9c, the deviation of *i *is expressed as follows:

V_o, a, b, i _= | S_b, i_(*o*) - S_a, i_(*o*) | + E (S_a, i_(*o*)), if S_a, i_(*o*) < S_a, b_(*o*) and S_b, i_(*o*) < S_a, b_(*o*)

    = | S_b, i_(*o*) - S_a, b_(*o*) | + E (S_a, i_(*o*)), if S_a, i_(*o*) > S_a, b_(*o*) and S_a, i_(*o*) > S_b, i_(*o*)

    = | S_a, i_(*o*) - S_a, b_(*o*) | + E (S_b, i_(*o*)), if S_b, i_(*o*) > S_a, b_(*o*) and S_b, i_(*o*) > S_a, i_(*o*).     (2)

E(x) = 0 if x is an integer, and E(x) = 0.5 if x is a half-integer. The total deviation W of a system is given by summing all possible quartets of *o*, *a*, *b*, and *i*.

W = ∑_i_W_i _= ∑_i _∑_o, a, b _V_o, a, b, i_.     (3)

### Core set approach

Our core set approach is achieved by an iteration of the following two procedures (a) and (b):

#### (a) Exclusion of sequences with deviations from the orthogonality

The first procedure identifies the sequence that gives the largest deviation (W_i _value in Eq. (3)), and then excludes it. By repeating this procedure until any W_i _values become zero, we obtain a P-core set of sequences that satisfies the orthogonality. The obtained core-set tree contains no correlated mutations and satisfies the compatibility. Therefore, it can be strictly reproduced by existing methods (MP, SP, ML and NJ). Each node sequence in this tree can be easily determined using three sequences which are of the nearest neighbour to this node.

#### (b) Insertion of excluded sequences based on a min-max criterion

The second procedure is to insert the excluded sequences into the P-core set tree, using a min-max criterion. By depositing each of these sequences into this tree, we can select a triplet (*o*, *a*, *b*) of probes that gives a maximum deviation, maxD, for this sequence, since the core set sequences satisfy the orthogonality perfectly. The sequence with the minimum value of maxD is the candidate. If PAUP* (HSEARCH with option of RANDOM) estimates a single MP tree, the expanded set becomes an updated core set and becomes the "current core set" in the next step. If not, the sequence is suspended in the remaining sequences and the sequence with the second smallest maxD becomes the candidate. Once the core set is updated, the maximum deviation from the updated core set becomes a new maxD values. This process continues as far as the core set is updated. As a result, we obtain the S-core set. We further expand the core set with the same process except that the tree of the current core set is regarded as real and fixed. If the phylogenetic location of the candidate sequence is uniquely determined by maximum parsimony, the sequence is added to the current core set resulting in the updated core set. Continuing this process until the core set cannot be updated, we obtain the T-core set. One insertion creates one node, the sequence of which is determined using the sequence to be inserted and the two node or endpoint sequences of the nearest neighbour. Here, the distances between this sequence and the other nodes and endpoints are modified so as to satisfy the additivity (W_i _= 0). Consequently, this sequence is identified as a core set member. The procedure of T-core set tree-building is so useful that the S-core set tree may be almost constructed by this procedure and finally confirmed by using the PAUP*. Software is available using Additional file 5.

#### (c) Local core sets to remove long-branch attractions

The first performance of the above-mentioned two procedures provides a "global core set" containing the maximum number of sequences for which MP has a single solution. However, attractions among long branches may make it difficult to further incorporate the excluded sequences into the global core set. To solve this problem, we decompose the global core set tree into two subgroups (each subgroup has to include more than three sequences). All the excluded sequences are merged into the first subgroup of core-set member and the procedure (a) is applied to the merged group (first subgroup + excluded sequence) with keeping a condition of preserving the tree structure of the global core-set. Then, the sequences that satisfy the orthogonality within this group are drawn out to construct a local P-core set. Here, when added sequences form the outside branches (outgroup) in this subgroup tree, they are suspended. We next insert the remaining excluded sequences by using the procedure (b), and get a local S-core set in which MP gives a unique tree within this subgroup. The two procedures are applied also to the second subgroup of core set. Here, the sequences inserted, as a result, into the two subgroups are suspended. The two procedures are applied to all possible pairs of subgroups. The procedure of T-core set tree-building is finally applied to the whole system for the remaining excluded sequences.

We furthermore decompose the global core set tree into more than three subgroups, and repeat the above-mentioned procedure. As a result, we obtain a maximum set of sequences in which there is no more sequence to be incorporated at a certain number of subgroups. It is here noted that MP proposes multiple solutions for the sequences containing the local S-core sets due to long-branch attractions.

#### (d) Remaining sequences with two phylogenetic positions.

The core set approach detected through practical applications that the remaining sequences despite the procedures (a)-(c) have two phylogenetic positions. The following three patterns were found for these unplaced sequences. Figure 7 shows three branching patterns that an ancestral sequence *1 *with a sequence of the same nucleotide (A, adenine) splits into three sequences of *2, 3 *and *4*. Here, A *n→*C denotes a substitution from A to C at the *n th *site. The dotted lines denote alternative positions of sequence *4*. One of two sites is shared in Fig. 7a. Two of three sites are shared in Fig. 7b. Two of four sites are shared in Fig. 7c. In this situation, sequence *4 *cannot be uniquely placed by the maximum parsimony criterion. However, since the alternative positions is explicitly indicated, we can estimate the nucleotide transitions in all possible branching patterns with the variance around their average value.

#### (e) CPU time of numerical calculations.

The majority of the CPU time is the calculation of the deviation index value, which includes the four DO-LOOPS in a FORTRAN program with double precision. This means that the CPU is proportional to N^4 ^with the number N of sequences. It took about 12 seconds for N = 36 sequences and 8 hours for N = 198 sequences with a standard personal computer (for instance, Pentium 2GHz). Therefore, the proportional constant is about 50000^-1 ^seconds. On the other hand, we used the PAUP* for the MP method, but this software has a limit (the number of sequences is less than 14) in the "exhaust" option in which all the possible topologies can be searched. Since it is well known that the number of topologies explodes with increase of N, our approach has an advantage in this respect.

## Authors' contributions

TO performed the majority of phylogenetic analysis and drafted the manuscript. YK, HK, TW, NN and YO performed some analysis of the data. SO and CW provided mosquito data, and assisted in biological interpretation. YK and HK participated in project coordination and the writing of the manuscript. All authors read and approved the final manuscript.

## Supplementary Material

Additional File 1 HIV-1 sequences and their accession numbers Click here for file

Additional File 2 The core set tree compared to the combined ML and MP. The topology was determined by ML using the 38 sequences of HIV-1, whereas the node sequences were obtained using MP. This tree pattern given by the two-step procedure broke the core set (Fig. 2a and 2b. Click here for file

Additional File 3 164 sequences used in Fig. 4b as one event of evolutionary simulation Click here for file

Additional File 4 The core set tree compared to the combined ML and MP. The topology was determined by ML using the 164 haplotypes of Fig. 4a, whereas the node sequences were obtained using MP. For simplicity, the branch pattern (a) of the two sub-groups 7–1 and 7–2 in Fig. 4a was compared with that given by the two-step procedure (b), in which the lineage of the *207th*, *242nd*, *284th *and *245th *haplotypes moved from group 7–2 to group 7–1. Click here for file

Additional File 5 A program file (GCS) for selecting the global core set sequences Click here for file
