# Supplementary figures and images for: Core set approach to reduce uncertainty of gene trees
Source: BMC Evol Biol. 2006 May 20;6:41. doi: 10.1186/1471-2148-6-41 (PMC1508163; doi:10.1186/1471-2148-6-41)

ML+PAUP\*

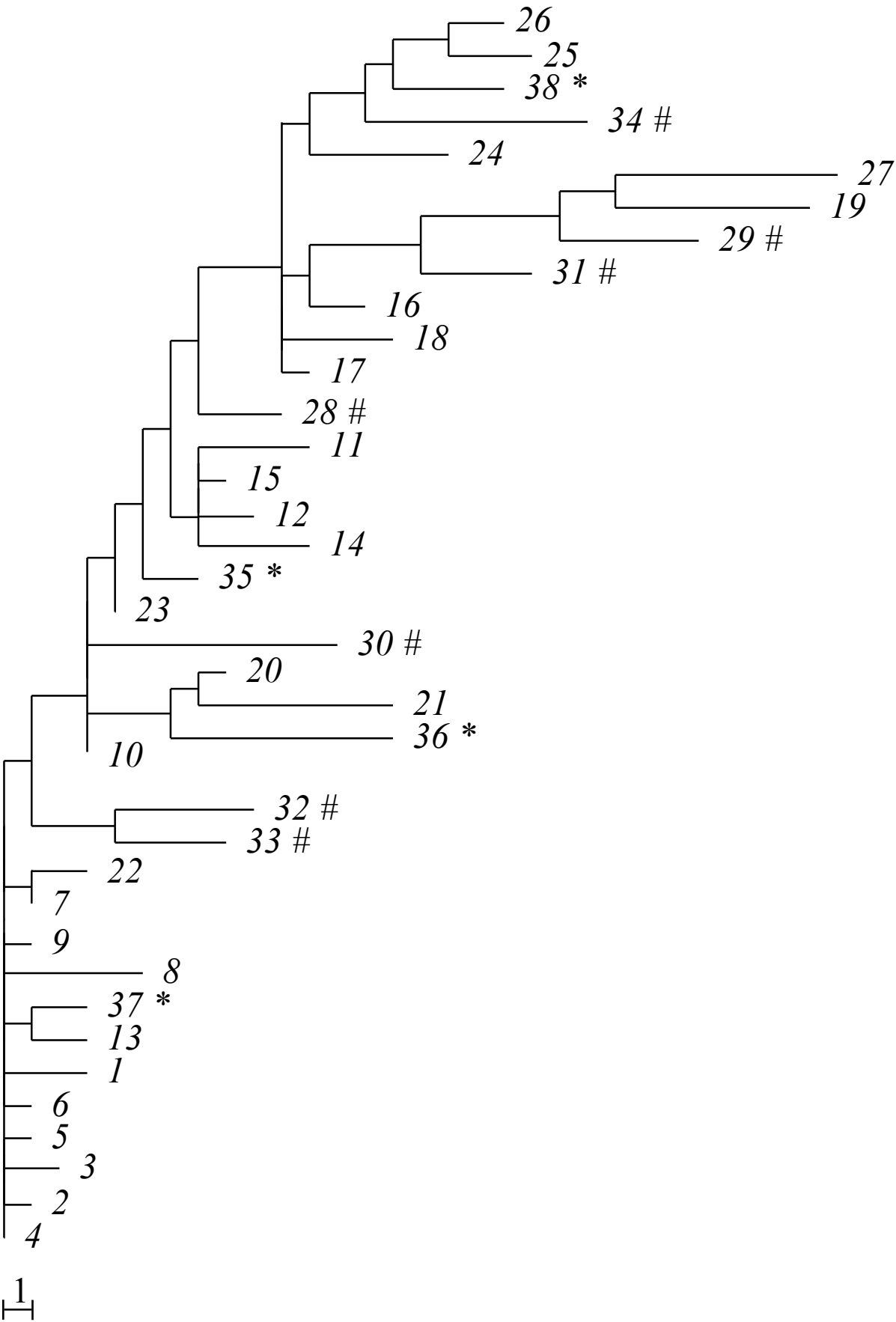

Supplement: Additional File 2 — The core set tree compared to the combined ML and MP. The topology was determined by ML using the 38 sequences of HIV-1, whereas the node sequences were obtained using MP. This tree pattern given by the two-step procedure broke the core set (Fig. 2a and 2b. [file 1471-2148-6-41-S2.pdf]

a (MVS)

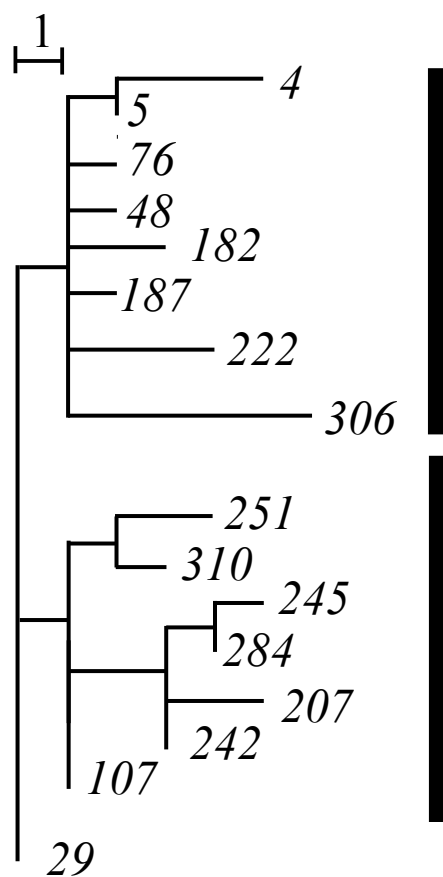

7-1

7-2

b (ML+ PAUP\*)

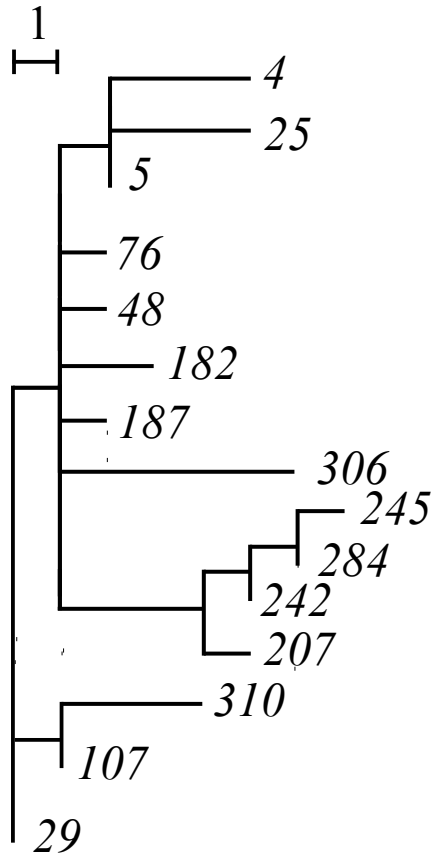

Supplement: Additional File 4 — The core set tree compared to the combined ML and MP. The topology was determined by ML using the 164 haplotypes of Fig. 4a, whereas the node sequences were obtained using MP. For simplicity, the branch pattern (a) of the two sub-groups 7–1 and 7–2 in Fig. 4a was compared with that given by the two-step procedure (b), in which the lineage of the 207th, 242nd, 284th and 245th haplotypes moved from group 7–2 to group 7–1. [file 1471-2148-6-41-S4.pdf]
